# Identification of serotypes of *Mannheimia haemolytica* and *Pasteurella multocida* from pneumonic cases of sheep and goats and their antimicrobial sensitivity profiles in Borana and Arsi zones, Ethiopia

**DOI:** 10.1038/s41598-023-36026-2

**Published:** 2023-06-02

**Authors:** Sisay Girma, Latera Getachew, Ayele Beyene, Desiye Tesfaye Tegegne, Takele Tesgera, Motuma Debelo, Jarso Debano, Dereje Teshome, Kebede Abdisa, Abebe Wirtu, Muluken Tekle, Birhanu Abera, Ketema Tafess, Morka Dandecha, Takele Abayneh, Belayneh Getachew, Takele Beyene Tufa, Teshale Sori Tolera

**Affiliations:** 1 College of Veterinary Medicine and Agriculture, Addis Ababa University, Bishoftu, Ethiopia; 2 College of Agriculture and Veterinary Medicine, Jimma University, Jimma, Ethiopia; 3 School of Veterinary Medicine, Ambo University, Guder, Ethiopia; 4 Yabello Regional Veterinary Laboratory, Oromia Agricultural Bureau, Yabello, Oromia Ethiopia; 5 Oromia Agricultural Research Institute, Yabello Pastoral and Dryland Agriculture Research Center, P.O. Box 85, Yabello, Ethiopia; 6 Department of Pharmacology and Toxicology, Wroclaw University of Environmental and Life Sciences, Wrocław, Poland; 7 National Veterinary Institute, Bishoftu, Ethiopia; 8 Asella Regional Veterinary Laboratory, Oromia Agricultural Bureau, Asella, Ethiopia; 9 Institute of Pharmaceutical Science, Adama Science and Technology University, P.O. Box 1888, Adama, Ethiopia; 10 Institute for Microbiology, University of Veterinary Medicine Hannover, Hannover, Germany

**Keywords:** Microbiology, Molecular biology, Medical research

## Abstract

Respiratory diseases caused by *Mannheimia haemolytica* (*M*. *haemolytica*) and *Pasteurella multocida* (*P. multocida*) have been known to result in a considerable loss due to mortality and reduced production. This study aimed at isolation and identification of *M. haemolytica* and *P. multocida* associated with pneumonic pasteurellosis in sheep and goats using bacteriological and molecular techniques. Identification of serotypes of *M. haemolytica* and *P. multocida* was done using indirect haemagglutination test. The in vitro antimicrobial sensitivity profiles of the *M. haemolytica* were tested using standard disk diffusion method. A total of 52 and 78 nasal swabs were collected from pneumonic cases for bacterial isolation and identification in Borana and Arsi zone, respectively. Four hundred sera samples were collected for identification of serotypes. The results showed that 17 of 52 (32.69%; 95% CI 20.33, 47.11) nasal swabs collected from pneumonic animals in Borana yielded positive results for *Pasteurella*/*Mannheimia* species, 13 (25.00%; 95% CI 14.03, 38.95) of which were *M. haemolytica*. None of the samples yielded *P. multocida*. Twenty-three of 78 (29.49%; 95% CI 19.69, 40.89) nasal swabs collected at Arsi from pneumonic animals yielded positive results for *M. haemolytica* (17) and *P. multocida* (6). Secondary biochemical characterization revealed that 14 of the 17 isolates conform to *M. haemolytica* whereas none of the 6 isolates suspected to be *P. mutocida* were confirmed. Eleven (84.62%) isolates from Borana and 4 (28.57%) from Arsi were confirmed to be *M. haemolytica* using PCR targeting the Rpt2 genes. Assay for *M. haemolytica* serotype A1 revealed all belong to A1. None of the isolates with cultural and morphological features of *P. multocida* gave positive results by molecular assay. Serological assay identified three serotypes of *M. haemolytica* namely A1, A2 and A7 almost in all of the samples whereas *P. multocida* serotype A was detected in 78.75% of the samples. The *M. haemolytica* isolates tested for susceptibility to antibiotics showed resistance against Bacitracin (83.33%) and Penicillin (50.00%) while they were found susceptible to Gentamycin (100%), Chloramphenicol (100%) and Sulfamethoxazole (100%) and Tetracycline (83.33%). In conclusion, the results of the present study revealed the association of *M. haemolytica* with pneumonic pasteurellosis in sheep and goats and can be of use in vaccine development in Ethiopia. Nevertheless, further investigations and continuous monitoring of antimicrobial resistance and appropriate selection and prudent use of antimicrobials in livestock sector are required.

## Introduction

Sheep and goats are very important assets for the Mediterranean, African, and Southeast Asian countries providing meat, milk, and wool. They are, however, known to be highly susceptible to respiratory diseases, which irrespective of the etiologic agents, account for about 50% of mortality and 5.6% of the total diseases among sheep and goats^1^. The prevalence and impact of respiratory diseases vary from country to country. For instance, in Australia, where extensive husbandry systems predominate, clinical respiratory disease in small ruminants is sporadic whereas, abattoir surveys in European countries suggest that the contribution of respiratory diseases to lowered productivity is considerable^2^. Although respiratory diseases are caused by multifactorial agents, certain serotypes of *Mannheimia haemolytica* (*M. haemolytica*) and *P. multocida* have been incriminated as primary bacterial agents in sheep and goats worldwide^3–6^. Respiratory diseases of sheep and goats usually result in economic losses arising from mortality and morbidity. The imminent impacts of respiratory diseases are mostly due to reduced production and mortality imposing considerable negative impacts on the industry.

Respiratory diseases of sheep and goats received huge veterinary attention in the European and North American situations^1^. Although published information is scarce regarding the incidence of respiratory disease in sheep and goats under Ethiopian conditions, previous reports revealed that they account for considerable mortality in sheep^7,8^ with an incidence of death ranging from 2.8 to 4.0% and case-fatality of 18%^8^. These previous authors claimed that the prevalence of morbidity and mortality due to pneumonic pasteurellosis was 49 and 36%, respectively. A review of the cases registered at the veterinary clinics between 2016 and 2021 in Borana pastoral zone, southern Ethiopia revealed that 27,563 and 37,522 cases of sheep and goats, respectively, of which 13,305 (48.27%) and 19,943 (53.15%) were pneumonia (preliminary data collected during this study). Similarly, in Basona district, 353, 522 and 106,441 cases of sheep and goats, respectively were recorded during the same period, of which 114,837 (32.48%) and 40,748 (38.28%) were pneumonia. These observations imply the significance of pneumonic pasteurellosis in small ruminants. However, reliable epidemiological data are lacking regarding the specific etiological agents involved and the identity number of strains selected for vaccine production. Few previous studies attempted to identify the serotypes of bacteria involved in field cases of pneumonic pasteurellosis of sheep and goats. For instance, the predominance of P. multocida serotype A, and M. haemolytica serotypes A1, A2, and A7 has been reported in goats in two districts in northern Ethiopia (Tanqua-Abergelle and Kola Tembien)^9^. Similar studies conducted by Berhe et al.^10^ showed the occurrence of these four serotypes in sheep and goats in Tanqua-Abergelle district. One study carried out in Wollo zone of north eastern Ethiopia revealed the occurrence of *M. haemolytica* A1, A5, A8, A9 and A13 in sheep^11^.

In Ethiopia only monovalent inactivated vaccine manufactured from *P. multocida* serotype A has been used for the control of pasteurellosis in sheep and goats. At the time when the vaccine was developed the objective was to control pasteurellosis caused by *P. multocida* in sheep and goats. In the field, however, veterinary practitioners often claim that the vaccine is used against any form of pneumonic pasteurellosis in sheep and goats. Empirical evidence showed that vaccinated flocks often succumb to pneumonic pasteurellosis. This could be due to the involvement of other serotypes than *P. multocida* serotype A. Selection of serotypes for vaccine production requires the understanding of the distribution of serotypes of high virulence and their association with diseases. Since the existing vaccine does not cover any of the serotypes of *M. haemolytica* research on the common serotypes associated with respiratory disease is important. The results can provide the vaccine producing companies with serotypes required for inclusion in vaccines and help veterinary practitioners in their informed decisions on vaccine choice. In this study, we identified the serotypes of *M. haemolytica* and *P. multocida* circulating in sheep and goats in Ethiopia using indirect haemagglutination test (IHAT). An attempt was also made to identify the species of bacteria associated with pneumonic cases of sheep and goats using conventional bacteriological methods and molecular techniques.

## Results

### Isolation and Identification of *M. haemolytica* and *P. multocida*

The result of presumptive identification of *M. haemolytica* and *P. multocida* is given in Table 1. Four hundred fifty-two sheep and goats were examined in the field in Borana pastoral zone (southern Ethiopia) for evidence of pneumonia of which 52 (11.50%; 95% CI 8.71, 14.81) were identified with clinical signs of pneumonia. Fifty-two nasal swab samples were collected from pneumonic animals and cultured for bacterial growth of which 17 (32.69%; 95% CI 20.33, 47.11) yielded positive results for *Pasteurella*/*Mannheimia* species. Phenotypic characterization using biochemical and morphological features revealed that 13 (25.00%; 95% CI 14.03, 38.95) of them were *M. haemolytica* whereas 4 (7.69%; 95% CI 2.14, 18.54) of them were *Bibersteinia trehalosi* (*B. trehalosi*). All of the 13 M*. haemolytica* isolates were isolated from sheep whereas 2 of the *B. trehalosi* isolates were from goats and the rest 2 from sheep. However, none of the samples gave positive results for *Pasteurella multocida*.

**Table 1:** Presumptive identification of *M. haemolytica* and *P. multocida* based on the secondary biochemical characteristics.

Phenotypic characteristics	Expected criteria	
	*M. haemolytica*	*P. multocida*
Haemolysis	+	−
MacConkey	+	−
TSI	+	−
Catalase	+	+
Indole	−	+
Arabinose	+	−
Maltose	v	+
Oxidase	+	+
Glucose	+	+
Sucrose	+	
Lactose	+	v
Motility	−	−
Urease	−	−
Citrate	−	−
Number of isolates presumptively identified	14	None

In Arsi zone (central Ethiopia) a total of 301 sheep and goats were examined and 78 (25.91%; 21.06, 31.25) of them had pneumonia. Seventy-eight nasal swab samples were collected from pneumonic animals and bacteria having cultural and morphological features of *M. haemolytica* and *P. multocida* were isolated from 23 (29.49%; 95% CI 19.69, 40.89) of them. Of these 17 (73.91%; 95% CI 51.59, 89.77) of them were phenotypically *M. haemolytica*. However, secondary biochemical characterization confirmed 14 of the 17 isolates to be *M. haemolytica*. Whereas 6 (26.09%; 95% CI 10.23, 48.41) of the isolates had cultural and morphological characteristics of *P. multocida* although none of them were found to be *P. multocida* by secondary biochemical characterization.

The result of frequency of isolation of *M. haemolytica* from nasal swabs collected from sheep and goats from the study sites is given in Table 2.

**Table 2:** The results of bacteriological culture on nasal swabs collected from pneumonic sheep and goats from the study areas.

Location	No. tested	No. positive	Proportion (%)
*Borana (Southern Ethiopia, Lowland)*			
Yabello	21	9	42.86
Dire	18	6	33.33
Gomole	13	2	15.38
Sub-total	52	17	32.69
Serotype identified		A1 (11/17)	64.71
*Arsi (Central Ethiopia, Highland)*			
D/Tijo	43	13	30.23
Tiyo	35	10	28.57
Sub-total	78	23	29.49
Serotype identified		A1 (4/23)	17.39
Overall total	130	40	30.77

### Results of molecular analysis

**Figure 1:**
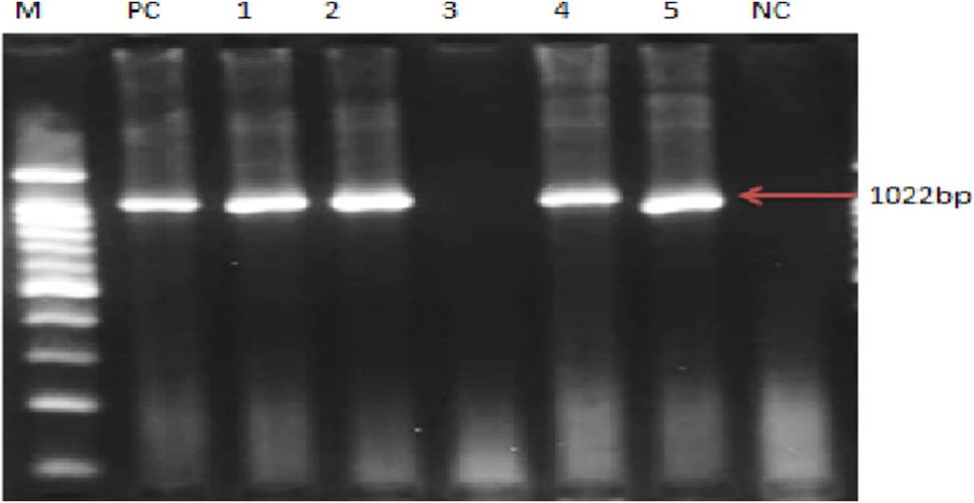
M = 100 bp molecular ladder; PC = positive control; Lanes 1–5 bacterial isolates tested in this study; NC = negative control.

Thirteen bacteria phenotypically identified as *M. haemolytica* from Borana were subjected to PCR targeting the *Rpt2* genes of *M. haemolytica* by mPCR. Eleven (84.62%) of the isolates were confirmed to be *M. haemolytica* using this method. Similarly, 14 of the isolates identified as *M. haemolytica* by bacteriological methods from Arsi were analyzed by the same molecular technique and 4 (28.57%) of them were confirmed to be *M. haemolytica* (Fig. 1). Serotype specific PCR assay showed that the *M. haemolytica* isolates belong to serotype A1. The isolates with cultural features of *P. multocida* were tested using molecular techniques but none of them gave positive results.

### Identification of serotypes of *M. haemolytica* and *P. multocida*

Out of a total of 400 sera samples assayed with IHAT three *M. haemolytica* serotypes namely serotype A1, A2 and A7 were identified in nearly all of the samples whereas *P. multocida* serotype A was detected in 78.75% of the samples (Table 3). Two hundred and ninety-eight of *M. haemolytica* A2 were identified in goats while 100 of them were detected in sheep sera. Three hundred and 99 of the *M. haemolytica* A1 were detected in goats and sheep, respectively. Similarly of the 399 sera yielding positive results for *M. haemolytica* A7 301 and 98 of them were from goats and sheep, respectively. Of a total of 315 samples giving positive results for *P. multocida* serotype A 235 and 80 of them were from goats and sheep, respectively.

**Table 3:** Serotypes of *M. haemolytica* and *P. multocida* identified in sera samples from sheep and goats using IHAT.

	Serotype			
	*M. haemolytica* A2	*M. haemolytica* A1	*M. haemolytica* A7	*P. multocida* A
No. tested	400	400	400	400
No. positive	398	399	399	315
Percent	99.50%	99.75%	99.75%	78.75%

### Results of antimicrobial susceptibility test

The antimicrobial sensitivity test showed that *M. haemolytica* developed resistance against Bacitracin (83.33%) and Penicillin (50.00%). In contrast, the *M. haemolytica* isolates identified in this study were found susceptible to Gentamycin, Tetracycline, Chloramphenicol and Sulfamethoxazole (Table 4).

**Table 4:** Results of antimicrobial susceptibility profile of *M. haemolytica* isolates against selected drugs.

Antimicrobial	Concentration	Resistant	Intermediate	Susceptible
Gentamycin	30 µg	0 (0.00%)	0 (0.00%)	6 (100%)
Tetracycline	10 µg	0 (0.00%)	1 (16.67%)	5 (83.33%)
Bacitracin	10 IU	5 (83.33%)	0 (0.00%)	1 (16.67%)
Sulfamethoxazole	25 µg	0 (0.00%)	0 (0.00%)	6 (100%)
Penicillin	10 IU	3 (50.00%)	0 (0.00%)	3 (50.00%)
Chloramphenicol	30 µg	0 (0.00%)	0 (0.00%)	6 (100%)

## Discussion

Control and prevention of pneumonic pasteurellosis in sheep and goats is an important endeavor to minimize the economic losses incurs to the small ruminant sector. Hence, understanding of the epidemiology of the disease including the catalogue of the etiologic agents is crucial. The current study demonstrated that *M. haemolytica* was associated with pneumonic cases in sheep and goats. The results revealed the significant role it plays in the pneumonia of sheep and goats in both highland (Arsi) and lowland (Borana) of Ethiopia although the involvement of other agents and the precipitating factors need to be elucidated. A noteworthy feature of the study is the identification of the serotypes of *M. haemolytica* and *P. multocida* using serological methods and the confirmation of the bacterial isolates with molecular techniques. The increased occurrence of pneumonic cases in sheep and goats as observed during the preliminary survey showed the extent of a respiratory problem that deserves investigation in the study areas. The generally stressful conditions in the study areas could possibly increase the probability of transmission of pathogens such as the occurrence of concurrent infections^8,23–25^. The dominant serotypes of *M. haemolytica* circulating among pneumonic cases in sheep and goats in the central highland and southern lowlands of Ethiopia were identified.

Bacteriological and molecular analysis did not reveal any *P. multocida* in this study although few of the nasal swabs yielded bacteria with morphological and cultural characteristics of *P. multocida*. *P. multocida* specific primers were used to confirm these isolates bu none of them gave positive results. However, a higher proportion of the sera samples tested showed the occurrence of antibodies against *P. multocida* serotype A. This could be due to the reaction of animals to the vaccine, which contains *P. multocida* serotype A. That is, these results suggest that the vaccine effectively reduced the occurrence of pneumonia caused by *P. multocida* in sheep and goats. In consent to our findings higher seroprevalence of *P. multocida* serotype A (60%) was observed in sheep in north Ethiopia^26^ and elsewhere in the world such as 83% in Spain^27^ and 87.5% in Egypt^28^.

In this study serological evidence showed that nearly all sheep and goats tested were found positive for *M. haemolytica* revealing the well-entrenched circulation of this bacterial species in the study areas. In consent to our observation, Berhe and colleague^10^ reported higher seroprevalence (98.7%) of *M. haemolytica* in goats in northern Ethiopia. We used molecular methods for confirmation of the *M. haemolytica* isolates, which have been shown to be an accurate, easy, available, and highly repeatable serotyping analogue in the field veterinary laboratories^29^. Only 55.56% of the isolates that were identified as *M. haemolytica* using bacteriological methods were confirmed by molecular methods. This shows the misidentification of these bacterial species using bacteriological (phenotypic) methods as there is no specific media or specific biochemical tests for *P. multocida* and *M. haemolytica*. The higher sensitivity and specificity of the molecular methods than the bacteriological techniques has been previously described^16^.

We confirmed the exposure of sheep and goats to serotypes of *M. haemolytica* considered having higher pathogenicity such as A2 and A1, which were reported to cause mortality in young lambs at feedlots elsewhere in the world^27^. Three serotypes of *M. haemolytica* namely A1, A2 and A7 are identified in samples collected from sheep and goats. Earlier studies conducted in Ethiopia also revealed that these serotypes were predominantly detected in sheep and goats. For example, Assefa and Kelkay^9^ observed that *M. haemolytica* serotype A1 and *M. haemolytica* serotype A2 were predominantly detected in goats in northern Ethiopia. Another study carried out in the same region revealed the predominance of *M. haemolytica* serotypes A1 and A2 in goats whereas *M. haemolytica* serotype A7 was more prevalent in sheep^10^. The reports of Sisay and Zerihun^11^ showed the occurrence of *M. haemolytica* serotypes A1 while that of Legesse and colleague^30^ revealed the predominance of *M. haemolytica* serotypes A1, A3, A5 and A9 in pneumonic sheep. A recent study in north Ethiopia also revealed the predominance of serotypes A7, A2 and A1 in sheep^25^. It has been shown in South Africa that *M. haemolytica* serotypes A1 and A6 were predominantly associated with pneumonia in sheep and goats^31^. Elsewhere in the world similar studies reported that *M. haemolytica* serotypes were isolated from 11.3% of clinical pneumonic cases of sheep predominated by A1, A2, A7^32,33^. Similarly the results of studies carried out by Gonzalez and colleague^27^ in pneumonic cases of sheep in Spain revealed the predominance of *M. haemolytica* A1, A2 and A12. In another study Arnal and colleague^6^ reported the predominance of *M. haemolytica* serotypes A1, A2 and A7 in sheep while *M. haemolytica* serotype A2 predominate in goats. The later authors also revealed that *M. haemolytica* serotypes A1, A2, and A6 were frequently associated with respiratory disease in ruminants. All these suggest the significance of *M. haemolytica* in causing pneumonic pasteurellosis in sheep and goats. The livestock authorities, researchers and vaccine producers should take this into account while planning prevention and control of respiratory diseases in sheep and goats.

The in-vitro antimicrobial sensitivity testing of *M. haemolytica* isolates showed 83.33% and 50.00% resistance against Bacitracin and Penicillin, respectively. The resistance against Penicillin observed in this study agrees with the reports of El-Seedy and colleague ^10,34^ in Egypt indicating the frequent use of this antibiotic in veterinary practices. Penicillin is an important antibiotic that has been widely used in human medicine and food-producing animals^35^ and this could have contributed to the development of resistance by *M. haemolytica* isolates against this antibiotic. The observed resistance by *M. haemolytica* against Bacitracin could be more related to the natural resistance mechanism which was mostly observed by other Gram-negative bacteria against cell wall inhibitors, like beta-lactam antibiotics; however, it needs further elucidation since Bacitracin is not a drug of choice to be used in sheep and goat health management practices in Ethiopia.

All of the *M. haemolytica* isolates tested were sensitive to Gentamycin, Chloramphenicol and Sulfamethoxazole and 83.33% of the isolates were sensitive to Tetracycline. The absence of resistance to Gentamycin could be due to the less frequent use in veterinary practices in Ethiopia although it is among the antimicrobials licensed for veterinary use^36^. Similarly, the 100% susceptibility of the organism to Chloramphenicol could be due to its limited usage in veterinary medicine as the drug is contraindicated to be used in food-producing animals and has not been used in livestock production in Ethiopia. However, in Ethiopia, oxytetracycline is the most widely prescribed antibiotic by veterinary professionals and the top misused drug by farmers to manage the health of livestock. The signal for tetracycline resistance in the current study (16.7%) is an alarm for its extensive usage in the study areas.

In conclusion, the results of the present study revealed the association of *M. haemolytica* with pneumonic pasteurellosis in sheep and goats and can be of use in vaccine development in Ethiopia. The vaccine to be developed and used should contain, at least, *M. haemolytica* serotypes A1 and A2, which are considered of high pathogenicity. Nevertheless, further investigations are required to obtain more information about *M. haemolytica* serotypes prevalent in sheep and goats in wider areas of the country, in order to design optimized control programs for pneumonic pasteurellosis. The isolates of *M. haemolytica* remain sensitive to most antimicrobials licensed for veterinary use. Continuous monitoring of antimicrobial resistance and appropriate selection and prudent use of antimicrobials by veterinary practitioners is important.

## Materials and methods

### Study areas

**Figure 2:**
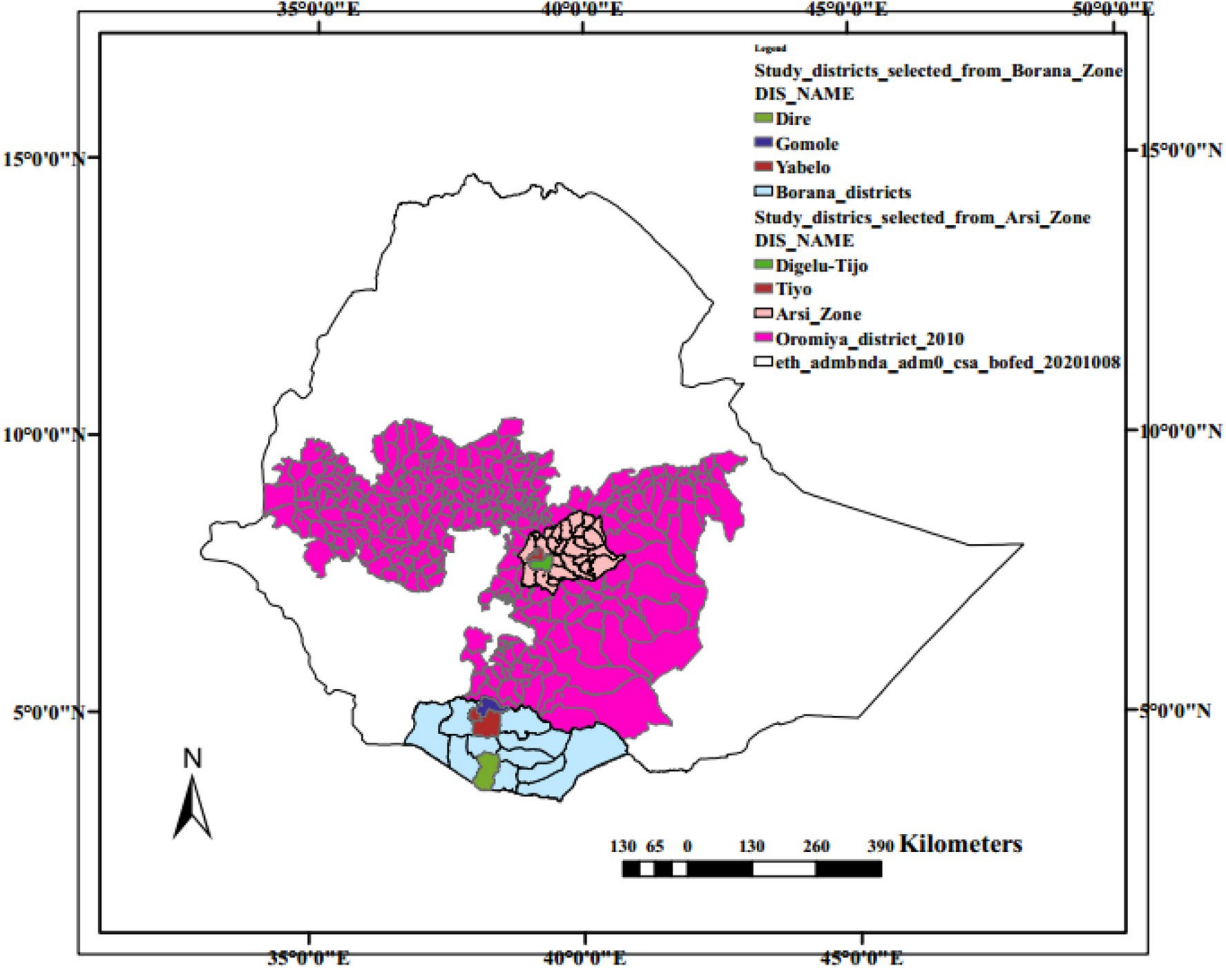
Map of Ethiopia depicting the geographical locations of the study areas.

The study was conducted in selected districts of Borana pastoral zone (southern Ethiopia) and Arsi zone (central Ethiopia), which were selected purposively (Fig. 2). Borana zone is a typical pastoral zone featured by livestock production in dominantly semi-arid and arid climate. Arsi zone, on the other hand, is one of the typical highland areas in central Ethiopia where mixed crop-livestock production is practiced.

Three localities namely Yabello, Gomole, and Dire were selected from Borana pastoral zone. The study areas are located in the southern part of Ethiopia between 3°36′–6°38′ North latitude and 3°43′–39°30′ East longitude. Yabello, which is the center of the zone, is situated at 570 km from the capital, Addis Ababa. The study districts receive an annual average rainfall ranging from 300 to 700 mm. The annual mean temperature of the areas varies from 19 to 24 °C. Livestock production (pastoralism) is the dominant economic activity in the areas. The mean number of sheep and goats per household in the areas is 9.5 and 13.8, respectively. The incidence of disease and mortality in sheep and goats is known to be high in this zone. The reports of the Central Statistical Agency^12^ for instance showed that 88,689 sheep and 88,502 goats were diseased during 2020/2021 of which 49,987 and 38,797, respectively died.

Arsi zone is located in Oromia Regional State at about 175 km away from the capital Addis Ababa. Two districts (Tiyo and D/Tijjo), which are found surrounding Asella town, the capital of the zone, were selected for this study. The districts have elevations ranging from 2400 to 3000 m above sea level. The average annual rainfall of the districts is 1658 mm whereas the mean annual humidity ranges from 43 to 60%. The annual mean temperature of the area ranges from 10 to 22.6 °C. The daily maximum temperature can reach up to 28 °C and the minimum temperature is as low as 10 °C. In both districts sheep and goat production play an important role in the livelihood of the farmers. This zone is also one of the zones in central Ethiopia where high incidence of disease and mortality has been recorded. During the 2020/2021 survey of the Central Statistical Authority 151,693 sheep and 104,965 goats succumb to diseases and 79,635 and 46,709 of them died^12^.

### Study animals and sampling

The study population consists of sheep and goats owned by pastoral community in the southern lowlands of Ethiopia (Borana zone) and smallholder mixed crop-livestock farmers in the central highland (Arsi zone). Borana pastoral zone and Arsi zone were selected purposively due to their agro-ecologies, the presence of a high number of sheep and goats, and the higher incidence of diseases and mortality as shown above. The study districts were also selected purposively for the same reason. Empirical evidence from the field showed that there has been frequent occurrence of respiratory diseases suggestive of pneumonic pasteurellosis in sheep and goats in the selected districts. Those sheep and goats brought to each district veterinary clinic were examined for the clinical evidence of pneumonia and those which were observed with anorexia, coughing, dyspnea, lethargy, bilateral nasal discharge, and abnormal lung sounds were considered to have pneumonia. Those pneumonic sheep and goats were considered in this study for bacteriologic and molecular characterization. In addition, sera samples were collected from apparently healthy sheep and goats for identification of the serotypes of *M. haemolytica* and *P. multocida* circulating in the study areas.

### Collection of nasal swabs

Nasal swabs were collected from those sheep and goats clinically diagnosed with pneumonia. Collection of the swab samples was conducted after appropriately restraining the animals with the assistance of veterinary personnel. The external part of the nostril was disinfected with 70% ethanol and sterile cotton-tipped, 20–25 cm long swabs were inserted into the nasopharynx via the ventral nasal meatus, rolled gently and pulled out. After sampling, the tips of each nasal swab were placed into individual tubes containing Amies transport medium (Oxoid, UK) and immediately transported on ice to the Yabello and Asella Regional Veterinary Laboratories.

### Isolation and phenotypic identification

The swabs were removed from the transport media and placed individually into brain heart infusion (BHI) broth (Oxoid, UK) and incubated aerobically at 37 °C for 6–8 h. A loop full of broth was streaked onto blood agar supplemented with 5% defibrinated sheep blood and MacConkey agar (Oxoid, UK) and incubated aerobically for 24 h at 37 °C. Growth of bacteria was monitored for *Pasteurella*/*Mannheimia*-like features such as colony color, motility, morphology after staining with Gram’s stain and haemolysis^13,14^. Those suspected colonies were sub-cultured onto nutrient agar and nutrient broth to perform primary (catalase and oxidase) and secondary biochemical tests as described by Quinn and colleague^15^. Presumptive identification was made on the basis of colony morphology, haemolysis, and Gram’s staining and biochemical tests. The biochemical characteristics examined include TSI, oxidase, indol, urease, citrate, catalase, and fermentation of glucose, lactose, sucrose, maltose, xylose and arabinose.

From the Phenotypically identified *M. haemolytica* and *P. multocida*, pure colonies were cultured onto slant nutrient agar in a screw cupped tube for 24 h at 37 °C and covered with sterile paraffin and stored at − 20 °C until transported to Holota Institute of Biotechnology for molecular characterization.

### Molecular detection and characterization

#### DNA extraction

For DNA extraction few colonies were taken from the pure cultures presumptively identified as *M. haemolytica* and *P. multocida* grown on blood agar for 24–48 h and transferred into 1.5 mL Eppendorf tubes. The genomic DNA was extracted using QIAGEN DNeasy Blood and Tissue Kit as per the manufacturer’s instructions (Qiagen, Germantown, MD, USA). The DNA purity was checked on 0.8% agarose gel electrophoresis and stored at − 20 °C until used.

#### PCR assay for detection of virulence associated genes of *M. haemolytica*

Species specific primers targeting Rpt2 gene coding for methyltransferase (Forward: 5′-GTT TGT AAG ATA TCC CAT TT-3′ and Reverse: 5′-CGT TTT CCA CTT GCG TGA-3′) were used in PCR assay as described in previous studies^16^. In brief, the PCR was carried out in a final volume of 25 μL of reaction mixture containing 10 μL of IQ Super mix (Bio Rad, USA) (DNA polymerase, dNTPs and buffer), 2 μL (5 pM/μL) of each primer pairs, 3 μL of RNase free water and 4 μL of template DNA. The PCR reaction was run at an initial denaturation at 95 °C for 3 min, followed by 35 cycles of each at 95 °C for 1 min, annealing at 48 °C for 1 min and extension at 72 °C for 30 s and a final extension cycle at 72 °C for 5 min. One reaction tube without the DNA template and the other with DNA template from reference *M. haemolytica* isolate from National Veterinary Institute culture collection (MH-NVI) were included as negative and positive controls, respectively. The primers were expected to amplify 1022 bp of the target gene.

### PCR assay for detection of *P. multocida*

For detection of *P. multocida* conventional PCR was carried out using primers designated KMT1T7-Forward (5′-ATC-CGC-TAT-TTA-CCC-AGT-GG-3′) and KMT1SP6-Reverse (5′-GCT-GTA-AAC-GAA-CTC-GCC-AC-3′) amplifying 460 bp of gene as described by Townsend and collegaue^17^. The PCR reaction was carried out in a final volume of 25µL containing 12.5 µL of master mix (Promega, USA), 1µL of primers (10 pM/μL), 3µL of DNA template and 7.5µL of RNAase free water. The polymerase chain reaction was performed on 'Prime' PCR with an initial denaturation of 95 °C for 3 min, followed by 35 cycles of denaturation at 95 °C for 1 min, annealing at 52.1 °C for 1 min, extension at 72 °C for 30 s, with the final extension at 72 °C for 7 min. The vaccine strain *P. multocida* obtained from National Veterinary Institute served as positive control and while one reaction tube without the DNA template was used as negative control.

Detection of the PCR products was done in 2% (w/v) agarose gel, prepared from 0.5X Tris borate EDTA buffer stained with Gelred. Each PCR product (5 μL) was mixed with 6X loading buffer and loaded into separate wells of the pre-prepared gel while 1 kb plus DNA molecular marker was loaded onto the first and last lane and run at 120 V for 60 min on electrophoresis apparatus (EC 2060, USA). The different band sizes of the PCR products were visualized under UV transilluminator and photographed in a gel documentation system (UVI TEC, UK).

### Identification of serotypes of *M. haemolytica* and *P. multocida*

About 5–10 mL of blood samples were collected aseptically from the jaguar vein of sheep and goats using a plain vacutainer tube and needle. The blood samples were transported to the nearby Regional Veterinary Laboratories where they were kept overnight at room temperature to allow clotting. The sera were then separated from the clot by centrifugation at 3000 g for 10 min and collected into labeled cryovials and stored at − 20 °C until transported to National Veterinary Institute, Bishoftu, Ethiopia where serotyping was carried out. Serotypes of *M. haemolytica* and *P. multocida* were identified by using the indirect haemagglutination (IHA) test as described by Biberstein and colleague^18,19^. Each serum sample was serotyped using the reference serotypes available in National Veterinary Institute as described below.

### Capsular antigen preparation

Briefly, the isolates of the *P. multocida* and *M. haemolytica* (PA, A1, A2, and A7) from NVI stock were allowed to grow on tryptose broth for 20 h at 37 °C. Full bacterial growth was monitored and the culture was centrifuged at 2000 g for 20 min. The sediment was resuspended with an equal volume of Phosphate Buffered Saline (PBS) and the suspension was heated in water bath at 60 °C and again centrifuged at 4500 g for 20 min at 4 °C. The clear supernatant was harvested and used as an antigen^20^.

### Sensitization of the antigen with sheep RBC

Blood was drawn from a jugular vein of a male sheep into a syringe containing Alsever’s solution and stored at 4 °C overnight and centrifuged at 2000 g for 10 min. The supernatant was discarded and 100 µL of RBC was added to 10 mL of the capsular antigens. This was followed by mixing of the RBC and capsular antigens with 50% glutaraldehyde. The mixture was homogenized by gentle agitation, incubated at 37 °C for 1 h and centrifuged at 2000 g for 10 min from which the supernatant was discarded. The sediment was resuspended with an equal volume of PBS and washed two times. Finally 10 mL PBS was added to the sediment to make a 1% suspension for use.

### Titration of antibodies

Ninety µL of PBS was dispensed into wells in the first raw of V-shaped microplate and 50 µL of PBS containing sensitized RBC was added to the rest of the wells including the negative and positive controls. Ten µL of sera were added to the wells in the first raw to get 1:10 dilution. After pipetting and mixing, 50 µL was transferred serially to the next wells and the last 50 µL was discarded. The plate was covered with a microplate, sealed and incubated at 37 °C with constant agitation for 1 h. The IHAT antibody titres of all the samples were recorded in comparison with the positive and negative controls. Positive results were taken if the level of antibody titer was greater than 1:10.

### Antimicrobial susceptibility tests

Antimicrobial susceptibility test was conducted on *M. haemolytica* isolates using the Kirby Bauer disk diffusion method^21^. The isolates were tested against six different antimicrobials with varied usage in veterinary practices in Ethiopia: extensively used (penicillin and tetracycline), commonly used (gentamycin and sulfamethoxazole) and rarely used (bacitracin and chloramphenicol). The interpretations were carried out according to CLSI^22^. Only six *M. haemolytica* isolates were tested against the selected antimicrobials due to the limited antimicrobial disc availability.

### Ethical approval

Ethical clearance for the use of study animals was obtained from Animal Research Ethical Review Committee of Addis Ababa University, College of Veterinary Medicine and Agriculture with the certificate reference number “VM/ERC/05/03/ 2021.

## Data Availability

The data used in this paper are available with the corresponding author.
